# Bacterial tryptophan metabolites in cancer and atherosclerosis: insights for a role in immune checkpoint inhibition

**DOI:** 10.1042/EBC20253060

**Published:** 2026-04-20

**Authors:** T Dormans, J Kroon, E Rampanelli, M Nieuwdorp, N van Es

**Affiliations:** 1 Department of Internal and Vascular Medicine, Amsterdam University Medical Center, Location AMC, Amsterdam, 1105 AZ, Netherlands; 2 Department of Experimental Vascular Medicine, Amsterdam University Medical Center, Location AMC, 1105 AZ, Amsterdam, Netherlands; 3 Amsterdam Institute for Infection and Immunity (AII), Amsterdam, Netherlands; 4 Atherosclerosis & Aortic Disease, Amsterdam Cardiovascular Sciences, Amsterdam, Netherlands; 5 Laboratory of Angiogenesis and Vascular Metabolism, VIB-KU Leuven Center for Cancer Biology, Leuven, 3000, Belgium; 6 Laboratory of Angiogenesis and Vascular Metabolism, Department of Oncology, KU Leuven and Leuven Cancer Institute (LKI), Leuven, 3000, Belgium

**Keywords:** amino acid metabolism, atherosclerosis, cancer, cardiovascular disease, coronary artery disease, immune response, immunology, inflammation, microbiome, therapeutics

## Abstract

The gut microbiota plays a pivotal role in human health, partly through the production of bioactive metabolites from dietary tryptophan. These indole derivatives have emerged as key modulators of immune function, inflammation, and metabolic health and have been linked to various diseases. In the context of cancer, indole derivatives are increasingly being studied as promising modulators of immune checkpoint inhibitor (ICI) therapy, with accumulating evidence indicating potential for various derivatives to enhance therapeutic efficacy. ICI therapy is associated with various immune-related adverse events, including accelerated progression of atherosclerotic cardiovascular disease. Given their immunomodulatory properties, there is a growing interest in the usage of indole metabolites to mitigate these cardiovascular complications. This mini-review summarizes current knowledge on the roles of microbiota-derived indoles in cancer, ICI therapy, and atherosclerosis. Though direct evidence linking bacterial tryptophan-derived metabolites to ICI-associated atherosclerosis is currently lacking, accumulating evidence indicates that indole derivatives regulate pathways involved in both anti-tumor immunity and atherosclerosis. Advancing our understanding of how the microbiome and its metabolites influence both cancer and cardiovascular disease will be crucial for developing personalized, metabolite-based strategies to improve outcomes in patients undergoing ICI therapy.

## Introduction

The human gut is a complex ecosystem harboring trillions of microorganisms, including bacteria, viruses, archaea, and eukaryotes, coined the gut microbiome. The majority of the microbial biomass is found in the cecum and proximal colon [1]. This dynamic microbial community plays a vital role in human health and is involved in various physiological processes including gut homeostasis [2], host immunity [3], and metabolism [4]. Therefore, perturbations in microbiome composition and consequent disruption of intestinal homeostasis can adversely affect these processes and have been associated with numerous disorders, such as autoimmune disease, type 2 diabetes, cancer, and cardiovascular disease [5].

In recent years, knowledge about the exact mechanisms by which specific gut microbiota contribute to disease has rapidly expanded. Research now not only focuses on the composition of the gut microbiome but also on its metabolic capacity that results in the production of various gut metabolites [6]. These microbial metabolites, including short-chain fatty acids (SCFAs) [7], bile acids [8], and various amino acid derivatives [9], play crucial roles in modulating host physiology and disease risk. Among these, tryptophan-derived metabolites, namely indole and its derivatives generated by gut microbiota, are increasingly recognized as crucial modulators of immune activity, inflammation, and metabolic health. Alterations in production of indoles, or shifts toward specific tryptophan degradation pathways, have been linked to a variety of metabolic and inflammatory disorders as well as malignancies including cancer [10].

In cancer therapy, a strong anti-tumor inflammatory response is pivotal for tumor remission. In this regard, immune checkpoint inhibitors (ICIs) have transformed cancer therapy by restoring T cell function through the blockade of inhibitory pathways that limit anti-tumor immune responses under chronic antigen exposure. ICIs are now a cornerstone in the management of malignancies such as melanoma, nonsmall cell lung cancer, and renal cell carcinoma. ICIs function by blocking key immune checkpoint molecules, including programmed cell death protein 1 (PD-1), programmed cell death ligand 1 (PD-L1), and cytotoxic T-lymphocyte associated protein 4 (CTLA-4), thereby enhancing the T cell-mediated anti-tumor responses [11]. Although the therapeutic benefit in cancer patients is evident, ICI therapy often leads to immune-related adverse events (irAEs) [12]. Most commonly, irAEs affect the gastrointestinal, endocrine, and dermatologic systems and may necessitate ICI discontinuation or temporary corticosteroid treatment, thereby reducing anti-cancer therapy efficacy [12,13].

**Figure 1:**
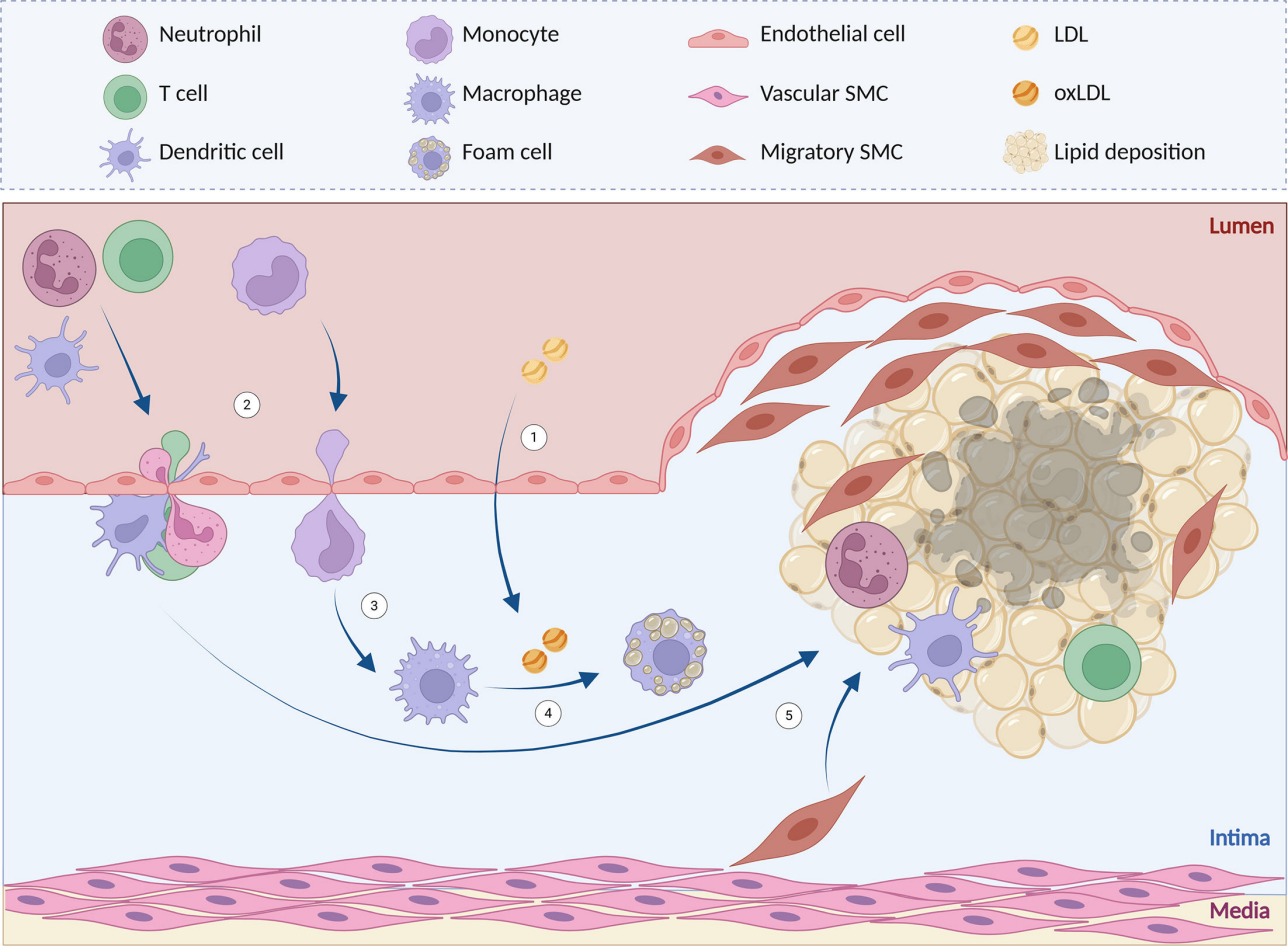
Atherosclerotic plaque formation.

Recently, ICIs have also been specifically associated with an increased risk of atherosclerotic cardiovascular disease (ASCVD), including myocardial infarction and ischemic stroke [14]. Atherosclerosis is a key driver of cardiovascular morbidity and mortality, is characterized by the formation of plaques in the vascular intima due to lipid accumulation and persistent low-grade inflammation (Figure 1) [15]. The mechanistic link between ICIs and atherosclerosis is thought to involve increased T cell activation and vascular inflammation, which can accelerate both the progression and destabilization of atherosclerotic lesions [14,16]—a phenomenon described in greater detail in Box 1 . Consequently, growing attention is being devoted to unraveling the pathophysiology of ICI-associated ASCVD [26,27], alongside the development of effective strategies for cardiovascular risk management in cancer patients [28].

Endothelial dysfunction and the accumulation of low-density lipoprotein (LDL) in the arterial intima promote the formation of oxidized LDL (oxLDL) (1), which elicits a potent inflammatory response and induces the expression of adhesion molecules such as vascular cell adhesion molecule 1 (VCAM-1) on endothelial cells. This facilitates the recruitment and transmigration of various immune cells, including circulating monocytes, from the lumen into the intima (2). Upon entering, monocytes differentiate into macrophages (3), which overtime form lipid-laden foam cells through the uptake of oxLDL (4). Sustained inflammation leads to foam cell necrosis and the development of a lipid-rich necrotic core. As the plaque evolves, further infiltration of immune cells and migratory smooth muscle cells amplifies local inflammation (5), promoting plaque progression and increasing plaque instability. Figure created using BioRender (https://www.biorender.com/).

**Box 1Immune checkpoint inhibitors and atherosclerosis**

Various imaging studies have demonstrated that ICI therapy contributes to atherosclerosis by promoting vascular inflammation. This aggravated inflammation has been shown to persist even beyond treatment discontinuation [16–18]. Since immune checkpoint molecules are present not only in the tumor microenvironment but also broadly expressed within the vasculature, therapeutic modulation of these checkpoints can influence the development and progression of atherosclerosis [19]. T cells are thought to be key contributors to ICI-associated atherosclerosis, as their therapeutically enhanced activity accelerates the advancement of atherosclerotic disease and promotes the formation of more unstable plaques, thereby increasing the risk of plaque rupture and acute cardiovascular complications [20]. This notion is supported by a recent systematic review and meta-analysis of murine studies, which found that plaques were predominantly composed of increased numbers of CD4^+^ T cells, CD8^+^ T cells, and macrophages [21]. In mouse models of atherosclerosis, anti-CTLA4 therapy can induce endothelial activation by increasing expression of adhesion molecules and can increase T cell activation and infiltration into the atherosclerotic plaque, resulting in plaque progression as well as decreased plaque stability [22]. The overexpression of CTLA4 in Apoe^−/−^ mice prevents atherosclerosis by suppressing dendritic and T cell activity [23]. Similar effects have been observed for the PD-1/PD-L1 axis, where knockout of PD-L1 in Ldlr^-/-^ deficient mice leads to an increase in atherosclerotic plaque burden, accompanied by increased T cell and macrophage infiltration and plaque instability [24]. Accordingly, promotion of the PD-1/PD-L1 axis decreases aortic plaque burden, reduces CD8^+^ T cell infiltration, and increases the presence of anti-inflammatory, IL-10–producing CD4^+^ T cells within atherosclerotic plaques [25].

Given the emerging recognition that gut microbiota-derived metabolites are important players in modulating the immune response and inflammation [10], indole metabolites have emerged as a promising avenue for optimizing both the efficacy and cardiovascular safety profile of ICI therapy. Elucidating the multifaceted roles of indole metabolites at the intersection of cancer, ICI therapy, and ASCVD may pave the way for novel therapeutic strategies and improved cardiovascular risk management in patients receiving ICI therapy.

## Tryptophan metabolism and microbiota-derived indoles

**Figure 2:**
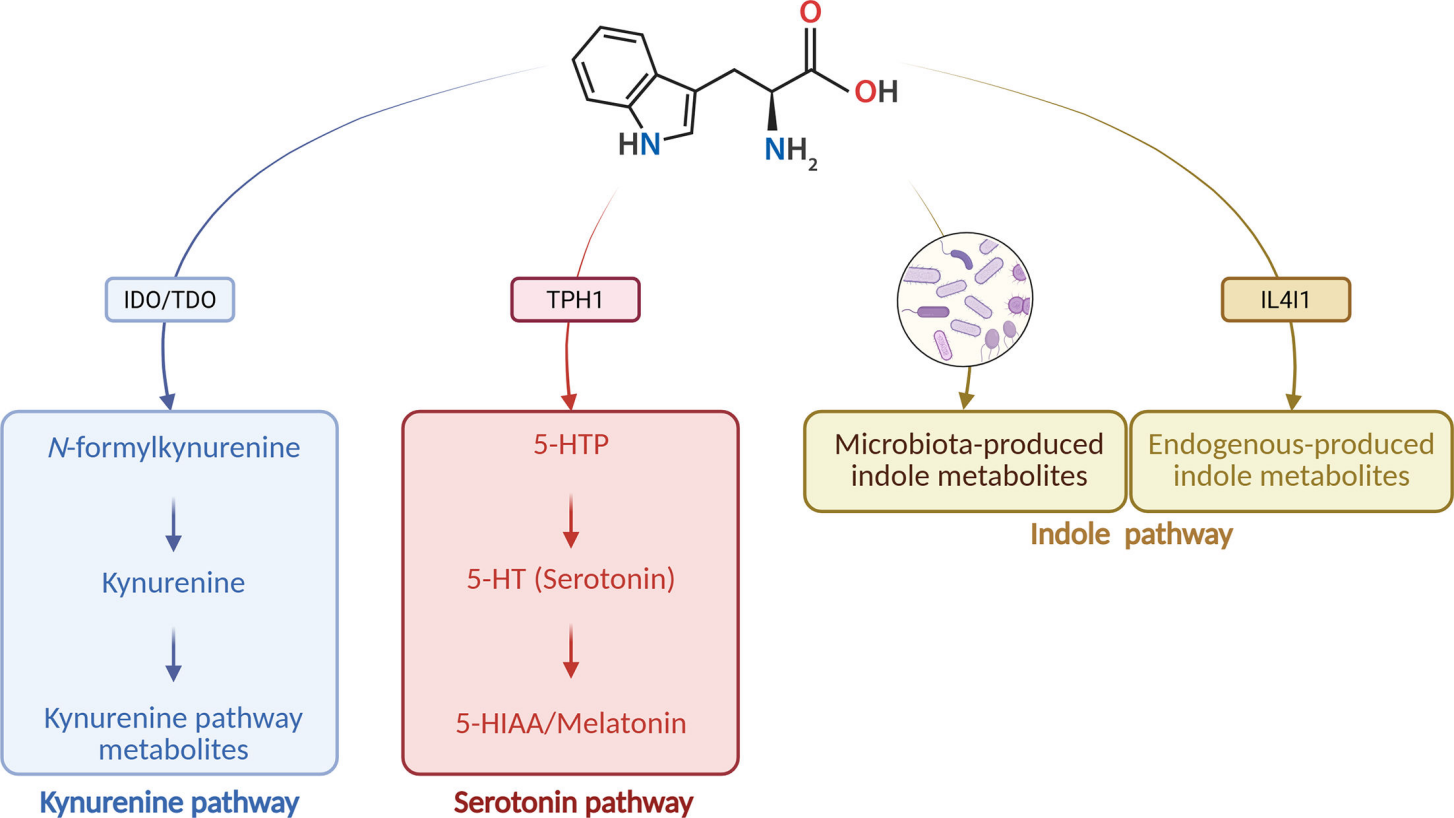
Overview of tryptophan metabolic pathways.

**Figure 3:**
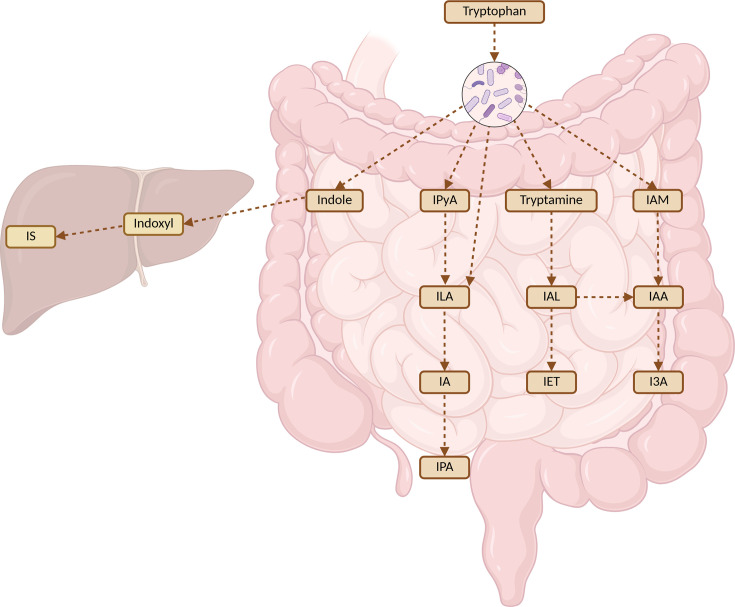
The indole pathway of tryptophan metabolism.

Tryptophan is an essential amino acid that must be acquired through the diet, as mammalian cells lack the enzymatic machinery for its *de novo* synthesis. Tryptophan serves as a precursor for several vital biomolecules that influence numerous physiological processes, including immune modulation, inflammation, and oxidative stress, making it crucial for maintaining immune homeostasis [10]. Tryptophan is metabolized via three distinct pathways: the kynurenine pathway, the serotonin pathway, and the indole pathway (Figure 2). The kynurenine pathway is the primary route, accounting for over 95% of tryptophan catabolism, and is catalyzed by the rate-limiting enzymes indoleamine 2,3-dioxygenase 1 (IDO1), IDO2, and tryptophan 2,3-dioxygenase 2 (TDO2). The serotonin pathway, responsible for producing serotonin or 5-hydroxytryptamine (5-HT), is mediated by tryptophan hydroxylase 1 (TPH1). Lastly, the indole pathway is primarily executed by gut microbial enzymes (Figure 3), although host-derived interleukin 4 induced 1 (IL4I1) can endogenously produce certain indole derivatives, such as indole-3-propionic acid (IPA) [10,29–31]. It is important to note that, in addition to the microbial indole pathway, both the kynurenine and serotonin pathway can also be influenced by gut microbiome composition, since certain bacteria possess the necessary enzymes for synthesis or modulate the expression of the mammalian enzymes involved in these pathways [32,33].

The breakdown of tryptophan occurs through three principal metabolic routes: (blue) the kynurenine pathway, mediated by indoleamine 2,3-dioxygenase 1 (IDO1), IDO2 and tryptophan 2,3-dioxygenase (TDO), leading to the production of N-formylkynurenine, kynurenine, and related metabolites; (red) the serotonin pathway, initiated by tryptophan hydroxylase 1 (TPH1), resulting in the synthesis of 5-hydroxytryptophan (5-HTP), serotonin (5-HT) and the downstream metabolites 5-hydroxyindoleacetic acid (5-HIAA) and melatonin; (yellow) the formation of indole derivatives, which can be produced by gut microbiota or endogenously by interleukin-4 induced-1 (IL4I1). Figure created using BioRender (https://www.biorender.com/).

Schematic representation of the indole pathway of tryptophan metabolism. IA, indoleacrylic acid; IAA, indole-3-acetic acid; I3A, indole-3-aldehyde; IAL, indole-3-acetaldehyde; IAM, indole-3-acetamide; IET, tryptophol; ILA, indole-3-lactic acid; IPA, indole-3-propionic acid; IPyA, indole-3-pyruvate; IS, 3-indoxylsulfate. Figure created using BioRender (https://www.biorender.com/).

Several genera, including *Bacteroides*, *Bifidobacterium, Clostridium,* and *Lactobacillus*, are capable of producing indole metabolites. The capacity to produce specific indole derivatives depends on the unique enzymatic repertoire of each microorganism [30]. Bacterial enzymes, such as tryptophanase, aromatic amino acid aminotransferases, and tryptophan decarboxylases, catalyze the conversion of tryptophan into a variety of indole derivatives, including indole-3-acetic acid (IAA), indole-3-propionic acid (IPA), indole-3-lactic acid (ILA), and indole-3-aldehyde (I3A) [29,30,34].

Indole and its derivatives play a pivotal role in maintaining immune homeostasis, primarily through the activation of the aryl hydrocarbon receptor (AhR), a ligand-activated transcription factor that integrates environmental signals, including dietary or microbial ligands, to shape metabolic and immune cell responses by transcriptional reprogramming [35]. Locally, tryptophan catabolism is largely involved in maintaining intestinal homeostasis by regulating immune responses and supporting proper epithelial barrier function. Systemically, these metabolites orchestrate the differentiation and function of various immune cell populations, thereby promoting immune tolerance and modulating inflammatory responses [10,30,36,37].

## Pro- and anti-tumorigenic effects of indoles

Various indoles have been linked to cancer, exerting effects primarily via AhR-receptor mediated signaling in immune cells. Several studies have reported pro-tumorigenic effects for various indoles. For example, in murine models of pancreatic cancer, dietary supplementation of IAA and ILA was shown to promote tumor growth by suppressing the anti-tumor immune response. Specifically, local breakdown of tryptophan by *Lactobacilli* in the gut to IAA and ILA induces AhR-dependent promotion of an anti-inflammatory phenotype in tumor-associated macrophages (TAMs) and suppresses CD8^+^ T cell cytotoxicity by inhibiting IFNγ expression in the tumor microenvironment, thereby facilitating tumor progression. Growth-promoting effects were not observed when dietary tryptophan was restricted and could be restored by oral gavaging of IAA and ILA [38]. Similarly, IPA derived from *Fusobacterium nucleatum* promotes tumor progression in colorectal cancer by inducing AhR activation in macrophages, thereby driving their polarization into anti-inflammatory M2-like macrophages and inhibiting CD8^+^ T cell numbers in the tumor microenvironment [39]. More pro-tumorigenic effects of indole metabolites have been reported in colorectal cancer models. For instance, trans-3-indoleacrylic acid (IDA), derived from *Peptostreptococcus anaerobius*, can promote tumorigenesis by inhibiting ferroptosis, a form of regulated cell death caused by iron-dependent lipid peroxidation. Indeed, IDA supplementation or supplementation of the IDA-producing bacteria *Peptostreptococcus anaerobius* in mouse models of colorectal cancer more than doubled tumor growth [40].

Conversely, anti-tumorigenic potential of certain indoles has also been demonstrated. In mouse models of colorectal cancer, oral supplementation with ILA-producer *Lactobacillus gallinarum* significantly reduced tumor growth by promoting apoptosis in colorectal cancer cells, an effect thought to be in part mediated by ILA [41]. Moreover, ILA produced by *Lactobacillus plantarum* can limit colorectal cancer progression in mouse models by enhancing the CD8^+^ T cell responses via epigenetic regulation [42]. Specifically, *in vitro* studies have demonstrated that ILA stimulates IL12a production in dendritic cells by increasing H3K27ac binding at the enhancer regions of the IL12a gene. Additionally, ILA induces repressive chromatin changes that support cholesterol metabolic fitness in CD8^+^ T cells [42], which is crucial for maintaining their functional capacity [43]. In contrast with the aforementioned pro-tumorigenic role for IPA, anti-tumor effects have also been reported. Through H3K27 acetylation at the TCF-1 enhancer (*Tcf7*), IPA promotes the activation of progenitor exhausted CD8^+^ T cells [44], a subset of T cells important for the long-term anti-tumor response [45].

Conflicting findings highlight both pro- and anti-tumorigenic effects of indole derivatives, suggesting that metabolite effects are highly context dependent. Interactions of indole metabolites with the tumor may vary substantially between and across tumor types, in part due to intrinsic tumor properties as well as immune, metabolic, and microbial differences in the tumor microenvironment [46,47]. Moreover, indole effects may differ depending on whether indoles are produced by gut microbes or locally by intra-tumor microbiota. For example, in a preclinical murine melanoma model, *Lactobacillus reuteri* was found to translocate into the melanomas and enhance T cell effector function in part through intratumoral production of I3A [48]. Effects on the tumor are not only metabolite and tumor microenvironment dependent, but are also influenced by the broader microbiome. The composition of both the gut and tumor microbiome is known to influence cancer outcomes. For example, in pancreatic cancer, the diversity of the local intratumor microbiome is an important determinant of long-term survival [49]. Further highlighting the importance of interaction within microbial communities, certain anti-tumor effects of indole metabolites are shaped not by individual taxa or metabolites but rather by interactions between bacteria species. The above-described anti-tumorigenic effects of IPA depend on the presence of the ILA-producing bacteria *Lactobacillus johnsonii*, as well as on the presence of *Clostridium sporogenes*, which converts ILA into IPA [44]. Cross-talk and interconnectedness between tryptophan pathways further add complexity to disentangling the (anti-)tumor effects of tryptophan metabolites. For instance, indole-3-carboxylic acid (I3CA), a metabolite produced by *Lactobacillus gallinarum*, can modulate the endogenous kynurenine pathway. I3CA can inhibit IDO1 in the tumor microenvironment, thereby limiting the production of kynurenine-AhR dependent promotion of tumor cell growth [50]. Such findings show that cross-talk of tryptophan pathways is an important factor to be considered when developing indole-based therapeutic strategies. This importance is further detailed in Box 2, which briefly outlines lessons learned from prior clinical therapeutic efforts targeting tryptophan metabolism.

**Box 2IDO1 inhibitors— - a lesson for indole-based therapeutic approaches?**

To date, clinical oncology studies of tryptophan metabolism have largely focused on the kynurenine pathway rather than indole-based interventions. Nevertheless, this prior clinical experience with kynurenine pathway inhibitors provides important insights for the development of indole-based strategies. Pre- and early clinical studies investigating the potential of IDO1 inhibitors to improve existing cancer therapies showed promising anti-tumor activity across various tumor types [51,52]. However, this initial enthusiasm was curbed when late-stage clinical trials did not demonstrate improved outcomes [53,54]. Various explanations have been proposed for the unexpected failure of these IDO1 inhibitors, including patient selection and suboptimal dosing [55,56]. In addition, an important role has also been found for metabolic adaptation, whereby IDO1 inhibition may trigger a shift towards a metabolic environment favorable to tumor survival, thereby potentially counteracting the anti-tumor activity of treatments [57–60]. For example, in ovarian tumors, IDO1 inhibition shifted tryptophan breakdown towards the serotonin pathway, thereby triggering NAD^+^-induced reduction in T cell functioning [56,57,59]. In melanoma, tryptophan depletion was an important contributor to anti-tumor immunity, and IDO1 inhibition restored tryptophan levels, thereby contributing to tumor survival [59]. Moreover, cancer therapies themselves may trigger activation of other tryptophan pathways, as illustrated by immunotherapy-induced activation of the IL4I1 pathway [58,61]. These discoveries have resulted in the exploration of novel inhibitory approaches, including the direct targeting of downstream AhR signaling [62–64]. Overall, these insights highlight that, for the successful development of indole-based strategies, careful consideration of tumor-intrinsic features, the tumor microenvironment, and potential metabolic adaptation is essential.

Taken together, indole derivatives can exert both pro- and anti-tumorigenic effects depending on the specific metabolite, tumor type, and the surrounding gut microbial ecosystem. This highlights the complexity and context-dependence of indole-mediated signaling in cancer progression and therapy. These findings underscore the necessity of evaluating indole metabolites within the broader context of microbial composition, metabolic pathways, and tumor environment.

## Indole metabolites in cancer immunotherapy

Given the reports on anti-tumorigenic effects, indole metabolites are increasingly being studied for their potential to enhance cancer therapy. Initially, beneficial effects have been reported in the context of chemotherapy. For example, in patients with pancreatic cancer, IAA levels were significantly associated with therapy efficacy, and IAA supplementation in mouse models increased chemotherapy efficacy [65].

Following these findings, attention has now turned to indole metabolites for optimizing immunotherapy, with increasing work investigating their potential to improve ICI efficacy and reduce toxicities. Recently, IPA was shown to be capable of improving the response to ICI therapy through activation of progenitor exhausted T cells. In both breast cancer and melanoma mouse models, IPA co-treatment with anti-PD-1 therapy greatly increased the number of progenitor T cells in the tumor microenvironment and significantly reduced tumor volume, whereas IPA treatment in the absence of anti-PD-1 exerted no significant changes [44]. In the aforementioned murine melanoma model where the I3A-producer *Lactobacillus reuteri* was found to translocate to the melanoma site, supplementation with either *Lactobacillus reuteri* or a tryptophan-rich diet significantly enhanced the effectiveness of ICI therapy [48]. In accordance, in stage IV melanoma patients, baseline serum I3A levels were not only significantly increased in ICI-responders but also associated with heightened progression-free survival and overall survival [48]. Enhancement of immunotherapy by indole derivatives can also be established through cross-activation of tryptophan pathways. For instance, I3CA secreted by *Lactobacillus gallinarum* inhibits tumor IDO1 and reduces immunosuppressive regulatory T cells (T_reg_) infiltration, thus improving anti-PD-1 efficacy in mice [50].

In addition to enhancing the efficacy of ICI therapy, indole metabolites may also hold potential in mitigating treatment-related toxicity. For example, in murine models of ICI-induced colitis - the most common gastrointestinal toxicity in ICI therapy - I3A treatment conferred protective effects against colitis. Interestingly, exemplifying the complex interplay between gut microbial pathways, chronic administration of I3A achieved this protective effect not only through modulation of the host AhR/IL-22 axis but also by shifting the gut microbial composition toward SCFA production [66], which is known to support intestinal barrier integrity, reduce inflammation, and hold anti-tumorigenic potential [7].

Evidence on the role of indole metabolites in ICI-associated cardiovascular toxicity remains limited to date. One study suggests IPA may offer cardioprotection in the context of anti-PD-1 treatment. In mouse melanoma models, treatment with leflunomide (Lef) - an immunomodulatory drug predominantly used for rheumatoid arthritis - increased IPA levels and greatly reduced anti-PD-1 associated cardiotoxicity whilst also increasing ICI efficacy. Lef treatment improved cardiac output, limited cardiac myocyte death, and impaired cardiac infiltration of CD8^+^ T cells. These effects were thought to be mediated by Lef-induced alteration of the microbial composition, causing an increase in IPA levels, thereby inducing cardiac AhR/PI3K signaling. This notion was supported by the observation that fecal microbiota transplants obtained from these Lef+anti-PD-1 treated mice could confer cardioprotective effects to other mice [67]. With respect to ICI-associated ASCVD, there is currently no direct evidence describing atheroprotective effects of indole metabolites. However, understanding the relationship between indole metabolites and ASCVD in a broader context may provide valuable insights for identifying potential atheroprotective strategies in an ICI context.

## Indole metabolites in atherosclerosis

Emerging data suggest an important role for indole metabolites in modulating ASCVD. Early clinical studies have reported inverse associations between various indole metabolite levels and ASCVD. In a cohort of patients with advanced ASCVD, plasma levels of indole, IPA, and I3A were significantly lower in patients with advanced atherosclerosis compared with controls, even after adjustment for traditional risk factors [68]. Conversely, heightened serum levels of IAA were found to be a positive predictor of cardiovascular events and mortality [69]. *In vitro* work in endothelial cells revealed that IAA can stimulate AhR-mediated inflammation through p38 MAPK/NF-*κ*B signaling and increase production of reactive oxygen species [69]. This atherogenic observation for IAA contrasts with the recent finding that IAA exerts broad atheroprotective effects; in Apoe^-/-^ mice, IAA supplementation reduced adhesion molecule expression on endothelial cells, limited inflammation *via* inhibition of the TLR4 pathway, and promoted anti-inflammatory M2-like macrophage polarization in atherosclerotic plaques [70]. Further supporting an atheroprotective role for IAA, studies found that supplementation with aucubin, a bioactive component often used in traditional Chinese medicine [71], altered the gut microbiota of Apoe^-/-^ mice, increased IAA levels through AhR activation and inhibition of TGF-β/Smad signaling [72]. The TGF-β/Smad pathway is a known driver of endothelial-to-mesenchymal transition and modulates vascular inflammation, plaque fibrosis, and atherosclerosis progression [73,74]. These findings suggest that the pro- or anti-atherosclerotic potential of IAA may be highly cell type-specific and therefore largely dependent on the experimental model used.

Despite these conflicting observations for IAA, mechanistic research for other indole derivatives largely points to an atheroprotective role for several indole metabolites. In Apoe^-/-^ mice, IPA supplementation was found to reduce plaque burden. In macrophages, IPA inhibits miRNA-mediated inhibition of the reverse cholesterol transporter ABCA1, thereby counteracting the detrimental accumulation of fat and cholesterols contributing to foam cell formation and atherosclerotic plaque development [75]. In a somewhat similar fashion, I3A can inhibit lipid accumulation in macrophages by upregulation of the miR-1271–5p, which in turn targets histone deacetylase 9, thereby increasing expression of ABCA1 as well as ABCG1 [76], both key atheroprotective transporters mediating cholesterol efflux [77]. Atheroprotective effects of I3A extend beyond macrophages, since, in endothelial cells, I3A can impair atherosclerotic development by reducing oxidative stress and decreasing expression of various pro-atherogenic factors including vascular cell adhesion molecule 1 (VCAM-1) [78], which plays a crucial role in atherogenic immune cell infiltration into the vessel wall [79].

As in cancer, interactions among tryptophan metabolism pathways can shape tryptophan metabolite generation in atherosclerosis. In Apoe^-/-^ mice, a high-fat diet can shift tryptophan metabolism away from gut microbial indole production toward the kynurenine and serotonin pathways, promoting dysbiosis, inflammation, and accelerated atherosclerosis. These atherogenic effects could be alleviated by indole metabolites, where supplementation of a mixture of IAA, IPA, and tryptamine significantly reduced plaque size [80]. Overall, these findings point toward predominantly anti-atherosclerotic effects of indole metabolites, which act by promoting anti-inflammatory conditions or via direct inhibition of inflammatory pathways.

## Conclusion and future perspectives

This mini-review has highlighted the diverse roles of microbiota-derived indole metabolites in cancer, ICI therapy, and ASCVD, as summarized in Table 1. As ICI therapy is associated with accelerated atherosclerosis and increased cardiovascular risk, there is an urgent need for strategies that protect against ICI-associated ASCVD without compromising anti-cancer effects. Indole-based interventions may represent a promising avenue, offering the potential to both enhance ICI therapy efficacy and mitigate the risk of atherosclerosis. However, a detailed understanding of the tumor microenvironment is required to anticipate how local factors may modulate the efficacy and safety of indole-based interventions. Moreover, when exploring the implementation of such approaches, the characteristics of the gut microbiome should be taken into account as microbes may utilize or further convert supplemented indole derivatives.

**Table 1:** Effects of indole derivatives on cancer immunity, ICI therapy, and atherosclerosis

Indole derivative	Cancer immunity			ICI therapy			Atherosclerosis		
	Reference cancer type	Main effect	Mechanisms	Reference cancer type	Main effect	Mechanisms	Reference	Main effect	Mechanisms
ILA	[38] Pancreatic cancer	Immune suppression and promotion of tumor growth	↑ Anti-inflammatory TAMs ↓ CD8^+^ T cell cytotoxicity						
	[41] Colorectal cancer	Reduction of tumor growth	↓ CRC cell proliferation ↑ CRC cell apoptosis						
	[42] Colorectal cancer	Reduction of tumor growth	↑ CD8^+^ T cell priming via IL12a production in DCs ↑ CD8^+^ T cell cholesterol metabolic fitness						
IAA	[38] Pancreatic cancer	Immune suppression and promotion of tumor growth	↑ Anti-inflammatory TAMs ↓ CD8^+^ T cell cytotoxicity				[69]	Increased inflammation and oxidative stress in endothelial cells	↑ AhR-mediated inflammation ↑ ROS ↑ p38 MAPK/NF-κB
	[65] Pancreatic cancer	Improvement of chemotherapy effect	↑ ROS accumulation ↓ Tumor metabolic fitness				[70]	Attenuation of atherosclerotic progression	↓ Endothelial cell adhesion molecules ↑ M2-like macrophages ↓ Aortic inflammation
							[72]	Reduction of endothelial inflammation and improved plaque stability	↑ AhR activation ↓ TGF-β/Smad
IPA	[39] Colorectal cancer	Promotion of tumor growth	↑ AhR activation in macrophages ↑ M2-like macrophages ↓ CD8 + T cells	[67] Melanoma	Improvement of ICI-induced cardiac dysfunction	↑ AhR-mediated PI3K signaling	[75]	Attenuation of atherosclerotic progression	↑ ABCA1-mediated reverse cholesterol transport in macrophages
	[44] Breast cancer, melanoma	Improvement of immunotherapy effect	↑ Progenitor exhausted T cell activation	[44] Breast cancer, melanoma	Improvement of immunotherapy effect	↑ Progenitor exhausted T cell activation	[68]	Inversely associated with advanced atherosclerosis	Not reported
IDA	[40] Colorectal cancer	Promotion of tumor growth	↓ Ferroptosis						
I3A	[48] Melanoma	Improvement of immunotherapy effect	↑ AhR signaling mediated CD8^+^ T cell effector functioning	[48 ] Melanoma	Improvement of immunotherapy effect	↑ AhR-signaling mediated CD8^+^ T cell functioning	[72]	Reduction of inflammation and lipid accumulation	↑ ABCA1, ABCG1 expression in macrophages ↑ M2-like macrophages
							[78]	Attenuation of atherosclerotic development	↓ Oxidative stress, ↓ VCAM-1 expression
				[66] Melanoma, lung cancer	Protection against ICI-induced colitis	↓ AhR/IL-22 axis and SCFA production	[68]	Inversely associated with advanced atherosclerosis	Not reported
IC3A				[50] Colorectal cancer	Improvement of immunotherapy effect	↓ IDO1 downregulation mediated CD8^+^ T cell functioning ↓treg differentiation			

Through both mechanistic and clinical studies, future research should elucidate the potential atheroprotective properties of indole metabolites in the context of ICI therapy. We hypothesize that supplementation of certain indoles or indole-producing bacteria may reduce ICI-induced accelerated atherosclerosis through the induction of a localized anti-inflammatory environment in the vasculature, while maintaining robust anti-tumor immunity. However, as some indoles may enhance CD8^+^ T cell activity and T cells are key mediators of ICI-associated atherosclerosis, the net effect of indole supplementation on vascular inflammation, atherosclerosis, and cancer therapy effectiveness remains uncertain and likely depends strongly on the specific metabolite, cancer type, and gut microbial composition. Unraveling the mechanisms by which indole derivatives influence ASCVD outcomes in an ICI context will not only deepen our understanding of host-microbiome interactions in the context of ICI therapy, but may also pave the way for microbiome-based interventions to mitigate cardiovascular risk in patients treated with ICIs.

On a broader scale, deciphering the intricate cross-talk between indoles and other gut metabolic pathways will be essential in expanding our comprehension of host responses in complex disease settings. An enhanced understanding of these interactions could transform the way we utilize microbiome- and metabolite-based therapeutic intervention. Ultimately, such insights could pave the way for microbiome-driven precision medicine at the intersection of cancer, atherosclerosis, and beyond.

**Summary Points**

- Various gut microbiota produce indole derivatives from tryptophan, which are key modulators of immune function and linked to various diseases including cancer and atherosclerosis.
- Indole metabolites can have both pro- and anti-tumorigenic effects, depending on the metabolite, tumor characteristics, and microbial composition.
- Some indole metabolites have shown potential to enhance the efficacy of immune checkpoint inhibitor (ICI) therapy. Certain indole metabolites, such as IPA and I3A, may protect against atherosclerosis by modulating inflammation and cholesterol metabolism, though findings for other metabolites like IAA are conflicting.
- Further research is warranted to explore the mechanisms and therapeutic potential of indole metabolites in preventing or reducing ICI-related atherosclerosis.

## Open Access

This article has been published open access under our Subscribe to Open programme, made possible through the support of our subscribing institutions, learn more here: https://portlandpress.com/pages/open_access_options_and_prices#conditional

## Abbreviations

ASCVD: atherosclerotic cardiovascular disease
AhR: aryl hydrocarbon receptor
CTLA-4: cytotoxic T-lymphocyte associated protein 4
5-HT: 5-hydroxytryptamine
I3A: indole-3-aldehyde
IA: indoleacrylic acid
IAA: indole-3-acetic acid
IAL: indole-3-acetaldehyde
IAM: indole-3-acetamide
I3CA: indole-3-carboxylic acid
ICI: immune checkpoint inhibitor
IDA: indoleacrylic acid
IDO1: indoleamine 2,3-dioxygenase 1
IDO2: indoleamine 2,3-dioxygenase 2
IET: tryptophol
ILA: indole-3-lactic acid
IL4I1: interleukin 4 induced 1
IPA: indole-3-propionic acid
IPyA: indole-3-pyruvate
IS: indoxyl sulfate
LDL: low-density lipoprotein
Lef: leflunomide
PD-1: programmed cell death protein 1
PD-L1: programmed cell death ligand 1
SCFAs: short-chain fatty acids
TAM: tumor-associated macrophages
TPH1: tryptophan hydroxylase 1
T_reg_: regulatory T cell
VCAM-1: vascular cell adhesion molecule-1
irAEs: immune-related adverse events
oxLDL: oxidized low-density lipoprotein
